# Cross-Species Differential Plasma Protein Binding of MBX-102/JNJ39659100: A Novel PPAR-*γ* Agonist

**DOI:** 10.1155/2008/465715

**Published:** 2008-09-14

**Authors:** Holly J. Clarke, Francine Gregoire, Fang Ma, Robert Martin, Spring Zhao, Brian E. Lavan

**Affiliations:** ^1^Research and Preclinical Development, Metabolex, Inc., 3876 Bay Center Place, Hayward, CA 94545, USA; ^2^Department of Molecular Biology, Genentech Inc., 1 DNA Way, South San Francisco, CA 94080, USA

## Abstract

Drug binding to plasma proteins restricts their free and active concentrations, thereby affecting their pharmacokinetic properties. Species differences in plasma protein levels complicate the understanding of interspecies pharmacodynamic and toxicological effects. MBX-102 acid/JNJ39659100 is a novel PPAR-*γ* agonist in development for the treatment of type 2 diabetes. Studies were performed to evaluate plasma protein binding to MBX-102 acid and evaluate species differences in free drug levels. Equilibrium dialysis studies demonstrated that MBX-102 acid is highly bound (>98%) to human, rat and mouse albumin and that free MBX-102 acid levels are higher in rodent than in human plasma. Interspecies differences in free drug levels were further studied using PPAR-*γ* transactivation assays and a newly developed PPAR-*γ* corepressor displacement (biochemical) assay. PPAR-*γ* transactivation and corepressor displacement by MBX-102 acid was higher in rat and mouse serum than human serum. These results confirm the relevance of interspecies differences in free MBX-102 acid levels.

## 1. INTRODUCTION

MBX-102/JNJ39659100 is a compound in development for the treatment of type 2 diabetes. It is a single enantiomer of halofenate, a drug that was tested clinically in the 1970s as a hypolipidemic and hypouricemic agent [1–6]. Although developed for lipid lowering, studies with halofenate in diabetic patients also demonstrated significant effects on plasma glucose and insulin both in monotherapy [7, 8] and in combination with other oral hypoglycemic agents [9–11]. Two decades later, it was discovered that both halofenate and MBX-102/JNJ39659100 are selective partial PPAR-*γ* agonists [12, 13] thereby offering an explanation for its antidiabetic properties.

Translational medicine is important for studying the action and safety of drugs. Studies in animals allow for interventional procedures that are not appropriate for humans. Key to interpreting these studies is to understand the relationship of the pharmacologically active form, (i.e., free drug) to the pharmacodynamic effects in each species studied.

Connecting preclinical pharmacology and safety studies in different species to the likely human experience therefore requires an understanding of the action of the drug at the target from these different species as well as the relationship of the free, pharmacologically active form to total drug concentration in these species.

For drugs with high serum protein binding this is particularly important. High serum protein binding appears to be a common feature of PPAR-*γ* agonists such as rosiglitazone, pioglitazone, and others [14–16] and previous data suggest that it may also be a feature of halofenate [17, 18] and therefore, also of MBX-102/JNJ39659100. Accurately determining free levels of highly plasma protein-bound drugs is technically challenging, making comparisons between species for these drugs extremely difficult. In the results reported herein, methods were used that allow for comparison between mouse, rat, and human plasma protein binding. This allowed for the appropriate interpretation of the pharmacology and potential for human risk of MBX-102/JNJ39659100. This study provides an approach that could be applied to the translational medicine and safety assessments for other PPAR agonists.

## 2. MATERIALS AND METHODS

**Figure 1:**
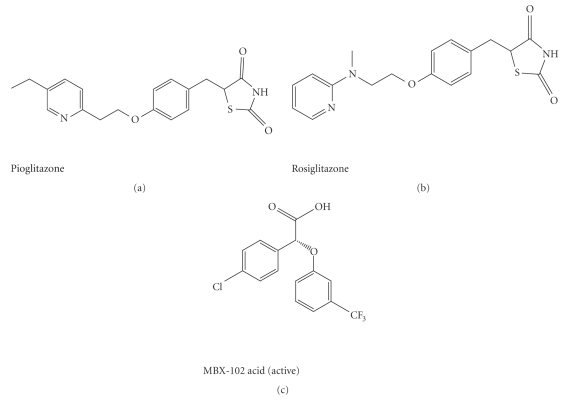
Structure of rosiglitazone, pioglitazone, and MBX-102 acid (active form).

[^3^H] MBX-102 acid (740 GBq/mmol, 20 Ci/mmol) was synthesized by Amersham Biosciences (Buckinghamshire, UK). MBX-102 acid was synthesized at IRIX Pharmaceuticals (Florence, SC, USA). The structure of MBX-102 acid is shown in Figure 1 in comparison to the full agonists, rosiglitazone and pioglitazone. For radio-labeled binding studies, pooled frozen plasma from either Sprague Dawley rats, CD-1 mice, or humans were purchased from Bioreclamation, Inc. (Hicksville, NY, USA). For the competitive equilibrium dialysis experiments, fresh pooled mixed gender plasma from either CD-1 mouse, Sprague-Dawley rat, or humans obtained from Bioreclamation, Inc. (Hicksville, NY) were used. Human, mouse, and rat serum albumins, and human alpha-1-acid glycoprotein were purchased from Sigma (St. Louis, Mo, USA). Charcoal stripped and delipidated sera from either human males, CD-1 male mice, or Sprague Dawley male rats were purchased from Biochemed (Winchester, Va, USA). FDG (Fluorescein di-*β*-D-galactopyranoside) was purchased from Invitrogen (Carlsbad, Calif, USA). Lanthascreen TR-FRET PPAR-gamma Coactivator Assay Kit and fluorescently labeled NCOR peptide (Fluor-DPASNLGLEDIIRKALMGSFDDK) were purchased from Invitrogen (Carlsbad, Calif). Steady Glo reagent was purchased from Promega (Madison, Wis, USA). DMEM culture media, Lipofectamine, Optimem, and Penicillin-Streptomycin were purchased from Invitrogen (Carlsbad, Calif). Bovine Insulin, isobutylmethylxanthine, and dexamethasone were purchased from Sigma (St. Louis, Mo). HEK 293T cells were obtained from ATCC (Manassas, Va, USA). Pro 293-Culture defined media was purchased from Cambrex (East Rutherford, NJ, USA).

### 2.1. Formulation of [^3^H] MBX-102 acid

 Radiolabeled MBX-102 acid was prepared as a 1 mL ethanol solution at a concentration of 50 *μ*M (1 mCi total). Stock MBX-102 acid dosing solutions (100-fold of final concentration) were prepared with unlabeled MBX-102 acid in dimethyl sulfoxide (DMSO) and spiked with 1 *μ*L/mL (0.05 *μ*M) of [^3^H] labeled MBX-102 acid so that the final evaluated concentrations of MBX-102 acid were 400 *μ*M, 600 *μ*M, 1000 *μ*M, 1500 *μ*M, and 2000 *μ*M. Final solvent concentrations were 1% of the total volume.

### 2.2. Determination of plasma protein binding of MBX-102 acid by equilibrium dialysis

Plasma was stored at −20°C. Prior to use, it was thawed and spun at approximately 2000 rpm for 5 minutes to remove any precipitated material. The pH was adjusted to pH 7.4 by careful addition of NaH_2_PO_4_. A 1 mL sample of spiked plasma was prepared by direct dilution of [^3^H]-MBX-102 acid stock solution into plasma and then added to one side of an equilibrium dialysis chamber. The other chamber was filled with 1 mL of 0.01 M phosphate buffered saline (PBS). The dialysis apparatus was placed in a water bath at 37°C and rotated at 20 rpm. Preliminary studies indicated that equilibrium is achieved within 5 hours (data not shown). Once equilibrium was established, the contents of the cell chambers were removed and analyzed by liquid scintillation counting. The chambers were sampled in triplicate. Nonspecific binding, in the absence of plasma, was determined to be 5.3 +/− 3.9% (mean +/− SD, *n* = 3). The mean recovery of [^3^H] MBX-102 acid was determined in triplicate by sampling of both dialysis chambers at each concentration of MBX-102 acid. The recovery percentage was found not to vary with MBX-102 acid concentration. The mean +/− SD % recoveries across all MBX-102 acid concentrations for each species were 83.9 +/− 6.7%, 84.4 +/− 2.4%, and 85.8 +/− 2.6% for human, rat, and mouse plasma, respectively.

### 2.3. Determination of protein binding of MBX-102 acid to selected human plasma proteins

Stock solutions of human serum albumin and alpha-1-acid glycoprotein were prepared in PBS buffer. Human serum albumin (40 mg/mL, ~600 *μ*M) and human alpha-1-acid glycoprotein (22.5 *μ*M) were spiked with [^3^H] MBX-102 acid. The spiked protein solution (175 *μ*L) was added to one side of an equilibrium dialysis chamber, and an equal volume of PBS buffer was added to the other chamber. Dialysis was allowed to reach equilibrium and the binding to protein was determined by liquid scintillation counting of samples from both chambers as described above. The percent recovery of [^3^H] MBX-102 acid with both serum proteins was between 95.7% and 98.5%.

### 2.4. Determination of MBX-102 acid binding to albumin by surface plasmon resonance (SPR)

The characterization of the binding of MBX-102 acid against human, mouse, and rat albumin was performed using SPR-based biosensors (Biosensor Tools, Salt Lake City, Utah, USA). The assay methods used to assess the binding of MBX-102 acid to human, mouse, and rat albumins have been described previously [19]. Briefly, each albumin was immobilized onto a CM5 sensor chip using standard amine coupling. Immobilization densities were between 10 000 and 13 000 RU. The test compound was run in a twofold dilution series with the highest concentration of 200 *μ*M. Each of the 16 different concentrations was tested in duplicate. The running buffer contained 53 mM Na_2_HPO_4_, 12.5 mM KH_2_PO_4_, 70 mM NaCl at pH 7.4, and 5% DMSO. All binding data were collected at 37°C. The binding response profile of MBX-102 acid over the three different albumin surfaces was evaluated and the binding constants for the high-affinity site were determined using a two-independent-site model. Conversion from K_D_ to %bound was performed as previously described [19].

### 2.5. Determination of species differences in protein binding of MBX-102 acid by competitive equilibrium dialysis

A comparison of the binding to plasma from different species was performed essentially by the method described below. Briefly, [^3^H] MBX-102 acid spiked plasma samples were formulated as described above with the exception that pH was not adjusted to 7.4 and the final DMSO concentration was 0.6%. A 1 mL sample of spiked human plasma was applied to one side of the dialysis membrane and 1 mL of spiked animal plasma was applied to the other side. The samples were dialyzed by rotation at 20 rpm for up to 120 hours in a 37°C incubator. The ratio of free drug in plasma was calculated according to the equation: ratio of free drug (animal versus human) = (total cpm in human plasma)/(total cpm in animal plasma).

### 2.6. Cell culture

HEK 293T cells (ATCC) were cultured in 15-cm dishes at subconfluence (approx. cell density was 14 000/cm^2^) in DMEM (high glucose), and 10% (v/v) fetal bovine serum (FBS) supplemented with 1% (v/v) Penicillin-Streptomycin. All cells were maintained at 37°C in a humidified atmosphere of 8% CO_2_ in air.

### 2.7. PPAR-*γ* reporter gene assays

HEK-293T cells were cultured as described above. Prior to use, the cells were trypsinized using 0.25% trypsin/1 mM EDTA and resuspended in DMEM, 10% (v/v) FBS lacking Penicillin-Streptomycin. For a pool sufficient to supply 100 wells, 6 million cells were diluted into medium for a total volume of 9 mL. The DNA-Lipofectamine 2000 mixture was prepared as per manufacturer’s instructions. For a pool sufficient to supply 100 wells, 5 *μ*g Gal 4-Mouse PPAR-*γ* LBD, 5 *μ*g pFR-Luciferase, and 500 ng Lac-z plasmids were mixed with 40 *μ*L of Lipofectamine 2000 in Optimem medium in a total volume of 1 mL. The cell suspension was mixed with 1 mL of the DNA-Lipofectamine 2000 mixture. The mixture was plated into a 96-well plate and incubated for 4 hours at which time the transfection medium was removed and replaced with 100 *μ*L DMEM, 10% (v/v) FBS and cultured overnight. The culture medium was then removed from the plates and replaced with 50 *μ*L Pro293A medium. Compounds and charcoal stripped/delipidated serum or serum albumin, *or* alpha-1 acid glycoprotein stock solutions were prepared at 2X final concentration in Pro293A medium and mixed together prior to addition of 50 *μ*L to the transfected cells and incubated for an additional 24 hours. Measurement of luciferase and fluorescence activity was performed according to the manufacturer’s instructions. Briefly, after removal of media, cells were incubated for 10 minutes in 100 *μ*L of Steady-Glo reagent. An 80 *μ*L lysate aliquot was transferred to opaque white well plates and the luminescence measured. The 80 *μ*L aliquot was then transferred back to the original plate. The fluorescence emission (excitation 485 nm, emission 535 nm) was measured after the addition of 100 *μ*L of 10 *μ*M fluorescein di-*β*-D-galactopyranoside in assay buffer (2.1 mM KH_2_PO_4_, 310.3 mM NaCl, 5.9 mM Na_2_HPO_4_-7H_2_O, 20 mM KCl, 2 mM MgSO_4_, 0.2% triton-X100). Each experimental condition was assessed in quadruplicate. The data were normalized for each well by dividing the luminescence measurement by the fluorescence measurement. Dose-response curves were generated and EC_50_ values were calculated using Prism Graphpad version 5.1.

### 2.8. Lanthascreen corepressor displacement assay

Assays were performed according to the manufacturer’s instructions. Briefly, GST-PPAR*γ*-LBD (5 nM), Tb-labeled anti-GST antibody (5 nM), and fluorescent-peptide (125 nM) were diluted together in kit assay buffer with 5 mM DTT and 10 *μ*L/well of this solution was added to 384-well black plates (Costar, Corning Inc. Life Science, Lowell, Mass, USA). Ligands were prepared as stock solutions in DMSO at 100-fold their final concentration followed by dilution to 2X concentration in kit assay buffer with 5 mM DTT containing a 2X concentration of serum albumin or charcoal stripped/delipidated serum prior to addition of 10 *μ*L/well to the assay plate. The plate was covered and incubated for 4 hours at room temperature. The time resolved fluorescence resonance energy transfer (TR-FRET) signal was measured using a Pherastar fluorescence counter (BMG labtech, Offenburg, Germany). The ratio of the emission intensity of the acceptor (Fluorescein: *λ* = 520 nm) divided by the emission intensity of the donor (Tb: *λ* = 490 nm) was then calculated to determine the degree of NCOR binding. Each measurement was performed in quadruplicate. Dose-response curves were generated and IC_50_ values were calculated using Prism Graphpad version 5.01.

### 2.9. Statistics

To compare logEC50 (or logIC50), ANOVA model of randomized block design was used. If block effect (experiment effect) was not significant, the data were reanalyzed by a reduced ANOVA model. Tukey’s test was used for multiple comparisons (SAS). Differences were considered significant at a *P* value <.05.

## 3. RESULTS

### 3.1. Interspecies protein binding of MBX-102 acid

MBX-102 is a selective partial PPAR-*γ* modulator which is structurally distinct from the full PPAR-*γ* agonists, rosiglitazone and pioglitazone (see Figure 1). In order to understand the relationship between free drug levels and the efficacy of the selective partial PPAR-*γ* agonist MBX-102 acid in different species, the plasma binding properties of MBX-102 acid were determined. Pooled, mixed sex plasma obtained from humans, Sprague Dawley rats, and CD-1 mice were spiked with MBX-102 acid and the % MBX-102 acid bound to protein was determined by equilibrium dialysis. The data shown in Table 1 reveal that MBX-102 acid is 99.5%–100% bound to plasma proteins from humans, rats, and mice. The high degree of binding observed was also independent of MBX-102 acid concentration. To identify potential MBX-102 acid binding proteins in humans, equilibrium binding studies were performed using purified human serum albumin and human alpha 1-acid glycoprotein. A high level of MBX-102 acid binding (>98%) to human serum albumin was observed. In comparison, the binding to human alpha 1-acid glycoprotein was very low (<5%) (data not shown). These studies indicate that the selective partial PPAR-*γ* agonist MBX-102 acid is highly protein-bound in plasma across different species and identifies serum albumin as a protein that binds MBX-102 acid.

To further characterize the binding of MBX-102 acid to albumin, we used surface plasmon resonance (SPR), a label-free technique that can be used to provide information on the kinetics and affinity of complex formation for drugs that are highly bound to albumin [19, 20]. The binding constants (KD) and the bound percentage for human, mouse, and rat albumin are reported in Table 2. In full agreement with the studies reported above, MBX-102 acid binding to albumin was >98%. This high degree of protein binding precluded any further analysis of differential binding of MBX-102 acid to plasma proteins across species because the absolute binding could not be determined accurately by any of the two methodologies used. Therefore, competitive equilibrium dialysis (CED) was used to address the question of differences in the binding of MBX-102 acid to plasma proteins among species. CED utilizes competition dialysis between the plasma of two species to accurately determine the ratios of the free drug fractions in these species [21]. Using this technique, the ratio of the free fractions is inversely related to the fold accumulation of total drug in the plasma of each species plasma at equilibrium. The ratios of rat-to-human and mouse-to-human free fraction were determined over several concentrations of MBX-102 acid. The data shown in Table 3 indicate that the free MBX-102 acid in rat plasma is 1.7 to 2.3 fold higher than in human plasma and that the free MBX-102 acid concentration in mouse plasma is 2.3 to 10.5 fold higher than in human plasma. Interestingly, both the rat-to-human and the mouse-to-human free drug ratios were found to decrease with total drug concentration possibly due to saturation of weak binding sites on human binding proteins. These findings predict that at a fixed total drug level of MBX-102 acid, the relative free drug levels across species will be in the order mouse > rat > human.

### 3.2. Activation of PPAR-*γ* by free drug in the presence of human serum

**Figure 2:**
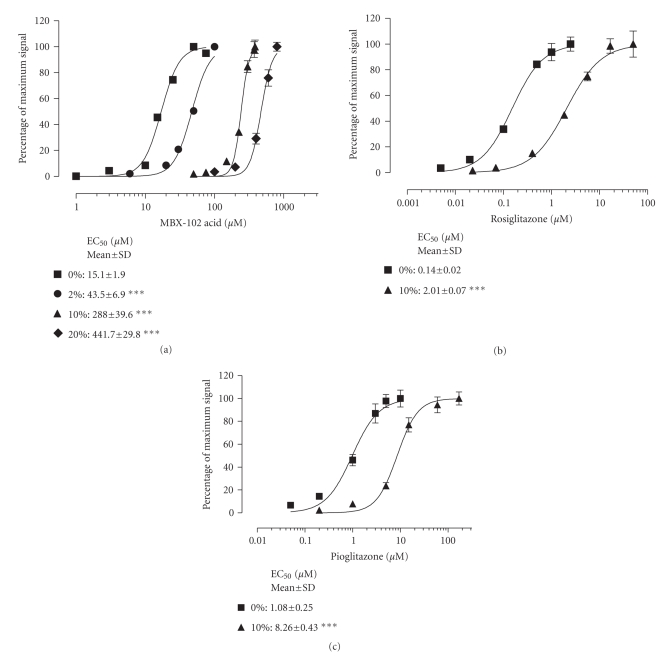
*PPAR-*γ* activation by (a) MBX-102 acid, (b) rosiglitazone, and (c) pioglitazone in the presence of increasing human serum.* Normalized reporter assay data were calculated as the percentage of maximum signal by expressing each data point as a percentage of the mean for the maximum signal. The percentage of maximum signal for the curves representing 0%, 2%, 10%, and 20% (v/v) serum was calculated independently. The dose-response curves shown are from a representative experiment. Values are EC_50_ (*μ*M) determined from 3 experiments and shown as the mean ± SD.

**Figure 3:**
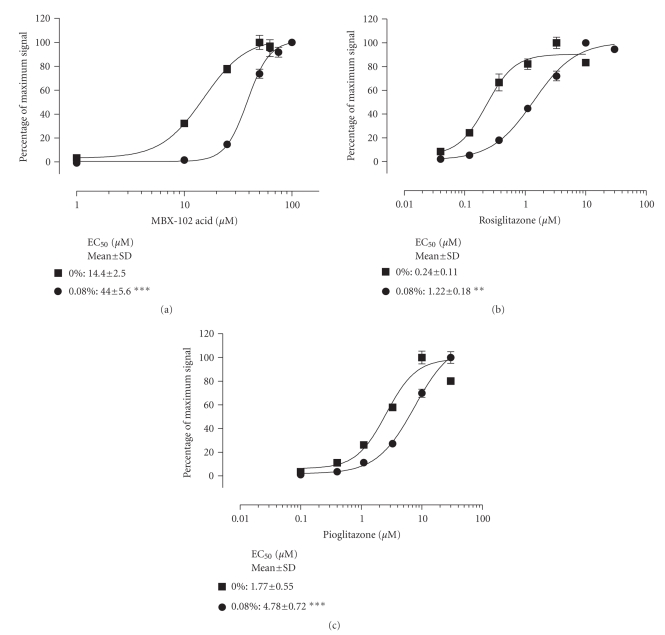
*PPAR-*γ* activation by (a) MBX-102 acid, (b) rosiglitazone, and (c) pioglitazone in the presence of increasing human serum albumin.* Normalized reporter assay data were calculated as the percentage of maximum signal as described in Figure 2. The percentage of maximum signal for the curves representing 0 and 0.08% serum albumin was calculated independently. The dose-response curves shown are from a representative experiment. Values are EC_50_ (*μ*M) determined from 2–6 experiments and shown as the mean ± SD.

The finding that the partial PPAR-*γ* agonist MBX-102 acid is differentially bound to plasma proteins across species suggested that the free levels, putatively responsible for pharmacodynamic effects of MBX-102 acid, could lead to a different dependence on total drug levels amongst the different species. In order to fully interpret the impact of different levels of free MBX-102 acid between species, it is essential to confirm that free drug level is responsible for the action at the receptor and to know if there are any intrinsic interspecies differences in PPAR-*γ* activity of MBX-102 acid. PPAR-*γ* reporter gene assays demonstrated that there were no intrinsic differences in the ability of MBX-102 acid to activate human, mouse, or rat PPAR-*γ* (data not shown). To understand the effect of serum on the activation of PPAR-*γ* by MBX-102 acid, the ability of MBX-102 acid to transactivate PPAR-*γ* was determined in a cell-based assay in the presence of increasing concentrations of human serum. As illustrated in Figure 2(a), MBX-102 acid induced PPAR-*γ* activity in a dose-dependent manner in the absence of serum. In the presence of increasing concentrations of human serum, there was a pronounced and serum concentration-dependent rightward shift of the dose-response curve for MBX-102 acid. The fold changes in mean EC_50_ values relative to no serum were 3, 19-, and 29-fold for 2%, 10%, and 20% human serum, respectively. At higher human serum concentrations, there was a decrease in the window of activation precluding an analysis of serum concentrations above 20%. Similar studies were performed for the full PPAR-*γ* agonists, rosiglitazone and pioglitazone (see Figures 2(b) and 2(c)). For both compounds, as was seen for MBX-102 acid, a rightward shift in the dose-response curve for PPAR-*γ* activation was observed in the presence of 10% human serum compared to serum free. For rosiglitazone, there was a 14-fold increase in EC_50_, and for pioglitazone, there was an 8-fold increase in EC_50_. Serum protein binding therefore affects the degree to which PPAR-*γ* can be activated by agonists in a cellular environment. Similar studies were performed for all three PPAR-*γ* agonists in the presence of human serum albumin. As expected, the EC_50_s for activation of PPAR-*γ* were rightward shifted in the presence of human serum albumin for all three PPAR-*γ* agonists (see Figures 3(a), 3(b), and 3(c)). Concentrations of serum albumin greater than 0.08% caused interference in the reporter assay precluding an analysis of the effect of higher and more physiologically relevant albumin concentrations. To further confirm the selectivity of the albumin effect, the EC_50_ for activation of PPAR-*γ* was also evaluated in the presence of alpha 1-acid glycoprotein. As anticipated, no shift in EC_50_ was detected even in the presence of the highest concentration of alpha 1-acid glycoprotein tested (0.14%, data not shown).

### 3.3. Differential activation of PPAR-*γ* across species

**Figure 4:**
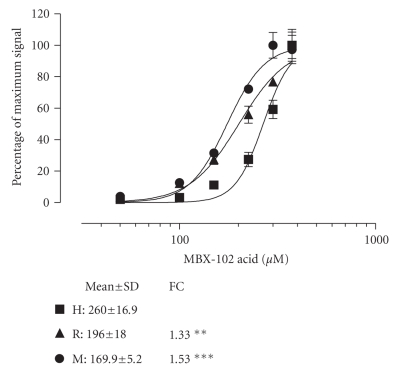
*Activation of PPAR-*γ* by MBX-102 acid in the presence of human serum compared to mouse and rat serum.* Normalized reporter assay data are expressed as the percentage of maximum signal as described in Figure 2. The dose-response curves shown are from representative experiments. MBX-102 acid activation of PPAR-*γ* in the presence of 10% (v/v) human (H), mouse (M), or rat (R) serum. The dose-response curves shown are from a representative experiment. Values are EC_50_ (*μ*M) determined from 3 experiments and shown as the mean ± SD. FC is the ratio of EC_50_s for human: rat or human: mouse (∗ = *P* < .05, ∗∗; = *P* < .01, ∗∗∗ = *P* < .001 by ANOVA with Tukey post hoc test).

On the basis of the finding that MBX-102 acid is differentially bound to serum proteins from human, mouse, and rat, and the confirmation that free drug levels determine the ability of MBX-102 acid to activate PPAR-*γ*, it is predicted that MBX-102 acid should differentially activate PPAR-*γ* in the presence of serum from different species. As illustrated in Figure 4, this was found to be the case. In the presence of 10% human, rat, or mouse serum, MBX-102 acid activated PPAR-*γ* with EC_50_s of 260 *μ*M, 196 *μ*M, and 170 *μ*M, respectively. These differences in EC_50_ were found to be highly statistically significant. Similar studies were also performed with the full PPAR-*γ* agonists, rosiglitazone and pioglitazone. As summarized in Table 4, MBX-102 acid activation of PPAR-*γ* was affected differently in the presence of 10% serum from different species compared to the effects seen with rosiglitazone and pioglitazone. For MBX-102 acid, the EC_50_ in the presence of mouse and rat serum occurred at lower concentrations than in human serum, whereas for both rosiglitazone and pioglitazone the opposite effect was observed, namely, that higher concentrations were needed in the presence of rat and mouse serum. These data suggest that the differential effect of serum on PPAR-*γ* activation observed with MBX-102 acid is a property of MBX-102 acid and not of the serum proteins.

### 3.4. Differential corepressor displacement from PPAR-*γ* across species

**Figure 5:**
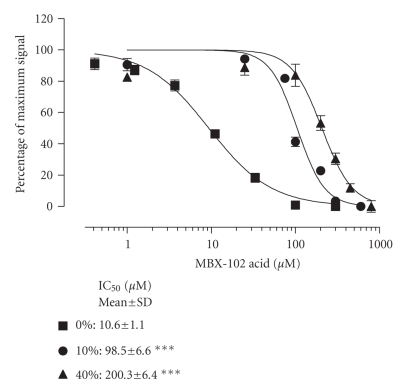
*Displacement of NCOR corepressor peptide from PPAR-*γ* by MBX-102 acid in the presence of human serum.* MBX-102 acid induced displacement of NCOR corepressor peptide from the human PPAR-*γ* ligand-binding domain in the presence of human serum at 0, 10%, or 40% (v/v). Normalized FRET assay data are expressed as the percentage of maximum signal (as described in Figure 2). The dose-response curves shown are from a representative experiment. Values are IC_50_ (*μ*M) determined from 3 experiments and shown as the mean ± SD.

**Figure 6:**
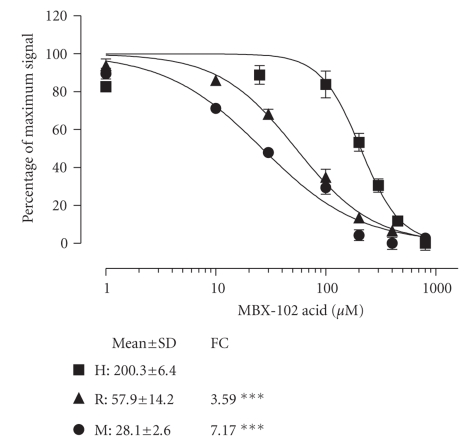
*
Displacement of NCOR corepressor peptide from PPAR-*γ* ligand-binding domain by MBX-102 acid in the presence of human serum compared to mouse and rat serum.* MBX-102 acid induced displacement of NCOR corepressor peptide from human PPAR-*γ* ligand-binding domain in the presence of 40% (v/v) human (H), mouse (M), or rat (R) serum. Normalized FRET assay data are expressed as the percentage of maximum signal (“percentage of maximal signal,” as described in Figure 2(a)). The dose-response curves shown are from a representative experiment. Values are IC_50_ (*μ*M) determined from 3 experiments and shown as the mean ± SD. FC is the IC_50_ fold change of mouse or rat compared to human (∗∗∗ = *P* < .001 by ANOVA with Tukey post hoc test).

The cell-based PPAR-*γ* reporter assay is adversely affected by mouse serum concentrations greater than 10% precluding analysis of cross-species differential serum binding at serum concentrations closer to physiological levels. An alternate in vitro assay was developed that allowed the assessment of the effect of much higher and more physiologically relevant serum concentrations on MBX-102 acid action. The data shown in Figure 5 demonstrate that a peptide derived from the corepressor NCOR is constitutively bound to the ligand-binding domain of PPAR-*γ* and can be fully displaced by MBX-102 acid with an IC_50_ of 11 *μ*M. Increasing concentrations of human serum caused a rightward shift of the dose-response curve resulting in up to a 19-fold shift in the IC_50_ at 40% human serum. Differential displacement of NCOR by MBX-102 acid was assessed at 40% serum for human, rat, and mouse (see Figure 6). The fold changes in IC_50_ for human-to-rat serum and human-to-mouse serum were 4 and 7, respectively. These data are very consistent with the relative free drug ratios predicted by the competitive equilibrium dialysis studies.

## 4. DISCUSSION

The data presented here demonstrate that MBX-102/JNJ39659100 is highly protein-bound, as had been suggested by previous studies with halofenate, and that at least one of the MBX-102 acid binding proteins is serum albumin. Our goal was to understand the serum binding properties of MBX-102 acid across species and to use this information in interpreting the pharmacodynamic and toxicological effects across species. The use of competitive equilibrium dialysis studies successfully demonstrated that MBX-102 acid is indeed differentially bound to plasma with the order of tightness of binding being human > rat > mouse. The studies performed using the cell-based PPAR-*γ* reporter assay confirmed, at least qualitatively, our hypothesis that the pharmacodynamic effects of MBX-102 acid are dictated by free drug levels and, further, that the differential binding of MBX-102 acid to serum proteins across species also results in a predictable and highly reproducible effect on pharmacodynamics. From these studies, the order of binding of MBX-102 acid to serum across species is predicted to be human > rat > mouse, which is in agreement with the data from the CED studies. Although we observed good qualitative correlations with the reporter assay and the CED assay, the magnitude of shifts in EC_50_ in the reporter assay was much smaller than those seen with the CED assay. One limitation of these reporter assay studies was the inability to investigate the effect of serum concentrations higher than 10% which could possibly explain the quantitative differences observed between these two assays. For this reason, we developed a new assay for measuring PPAR-*γ* activity in vitro that was able to tolerate serum concentrations as high as 40%. The data from this new assay confirmed the predicted order of binding for MBX-102 acid to serum across species as human > rat > mouse and also provided quantitatively very similar fold changes to the CED assay. The basis of the differential binding of MBX-102 to serum albumin from different species is unknown. Although at the protein level, mouse and rat albumins are highly conserved (~90% homology), the degree of conservation is much lower between human and mouse (~72%) and human and rat (~73%). Such differences may, at least in part, be responsible for the differential binding observed between species.

The approaches described here will be generally useful for interpreting preclinical pharmacology data in different species as well as toxicology studies and how these will relate to the human experience. Whilst confined initially to PPAR-*γ*, the approaches could easily be adapted for PPAR-*α* and PPAR-*δ* and indeed to virtually any other ligand-modulated receptor.

## Figures and Tables

**Table 1:** *Binding of MBX-102 acid to rat, mouse, and human plasma determined by equilibrium dialysis.* Binding of [^3^H] MBX-102 acid to plasma was conducted by equilibrium dialysis against PBS buffer at 37°C and the percentage of total radiolabel bound to plasma was determined by dividing the amount of sample in the plasma compartment by the combined total amounts in the plasma and PBS buffer compartments. Values represent the result of a representative experiment and are the mean ± SD of triplicate determinations.

MBX-102 acid (*μ*M)	%Protein Binding ± SD		
	Human	Mouse	Rat
400	99.8 ± 0.1	99.8 ± 0.0	99.8 ± 0.1
600	99.8 ± 0.1	99.7 ± 0.0	99.8 ± 0.1
1000	99.7 ± 0.1	99.5 ± 0.1	99.7 ± 0.0
1500	100 ± 0.1	99.8 ± 0.1	99.8 ± 0.2
2000	99.8 ± 0.1	99.5 ± 0.1	99.5 ± 0.1

**Table 2:** *Binding of MBX-102 acid to rat, mouse, and human albumin determined by plasmon resonance-based biosensors.* The binding constants for the high-affinity site were determined at 37°C. Values represent the mean of duplicate determinations (HSA: human serum albumin, MSA: mouse serum albumin, RSA: rat serum albumin).

Interaction	K_D_ (*μ*M)	%Bound
HSA:MBX-102	5.8	99.1
MSA:MBX-102	5.5	99.2
RSA:MBX-102	12.8	98.1

**Table 3:** *Interspecies free MBX-102 acid ratios determined by competitive equilibrium dialysis*. [^3^H] MBX-102 acid distribution between either mouse and human plasma or rat and human plasma was conducted by competitive equilibrium dialysis at 37°C. Values represent mean ± SD for 5 independent experiments.

MBX-102 Acid (*μ*M)	Free Fraction Ratio (*n* = 5 ± SD)	
	Rat:Human	Mouse:Human
100	2.3 ± 0.6	10.5 ± 5.5
300	2.3 ± 0.6	5.9 ± 3.6
700	2.0 ± 0.3	3.7 ± 1.9
1000	1.8 ± 0.2	2.6 ± 1.1
1300	1.7 ± 0.2	2.3 ± 0.7

**Table 4:** *Differential activation of PPAR-*γ* by PPAR-*γ* agonists in the presence of 10% of human, rat, and mouse serum.* Values are EC_50_ (*μ*M) determined from 3 experiments and shown as the mean ± SD. FC is the ratio of EC_50_s for human: rat or human: mouse (∗ = *P* < .05, ∗∗ = *P* < .01, ∗∗∗ = *P* < .001 by ANOVA with Tukey post hoc test).

PPAR agonist	Mean EC_50_ (*μ*M) ± SD			Fold Change in EC_50_	
	Human	Rat	Mouse	Human:Rat	Human:Mouse
MBX-102 acid	260 ± 16.9	196 ± 18	169 ± 5.2	1.33**	1.53***
Rosiglitazone	2.0 ± 0.1	5.2 ± 0.3	4.5 ± 0.3	0.39***	0.45***
Pioglitazone	8.3 ± 0.4	11.4 ± 1.2	9.7 ± 1.4	0.73^NS^	0.86^NS^
